# Development of β-Cyclodextrin/Konjac-Based Emulsion Gel for a Pork Backfat Substitute in Emulsion-Type Sausage

**DOI:** 10.3390/gels8060369

**Published:** 2022-06-11

**Authors:** Yea-Ji Kim, Dong-Min Shin, Jong-Hyeok Yune, Hyun-Su Jung, Hyuk-Cheol Kwon, Kyung-Woo Lee, Jae-Wook Oh, Beob-Gyun Kim, Sung-Gu Han

**Affiliations:** 1 Department of Food Science and Biotechnology of Animal Resources, Konkuk University, Seoul 05029, Korea; dpwl961113@konkuk.ac.kr (Y.-J.K.); s900704@konkuk.ac.kr (D.-M.S.); skyun0423@konkuk.ac.kr (J.-H.Y.); jehceh12@naver.com (H.-S.J.); rnjs1024@konkuk.ac.kr (H.-C.K.); 2 Department of Animal Science and Technology, Konkuk University, Seoul 05029, Korea; kyungwoolee@konkuk.ac.kr (K.-W.L.); bgkim@konkuk.ac.kr (B.-G.K.); 3 Department of Stem Cell and Regenerative Biotechnology, Konkuk University, Seoul 05029, Korea; ohjw@konkuk.ac.kr

**Keywords:** β-cyclodextrin, konjac, emulsion gel, fat substitute, low-fat sausage

## Abstract

Emulsion gel has been used to replace animal fats in meat products. Konjac is a widely used gelling agent; however, its low emulsion stability limits its use in meat products. This study aimed to examine the quality characteristics of β-cyclodextrin (CD)-supplemented konjac-based emulsion gel (KEG) (CD-KEG) and its application as a fat substitute in emulsion-type sausages. The supplementation of CD increased hydrogen bonds and hydrophobic interactions with konjac and oil in the gels, respectively. Additionally, CD increased the structural complexity and strength of KEG. Since adding more than 6% of CD to KEG did not increase the gel strength, 6% CD-added KEG was adopted to substitute for pork backfat in manufacturing low-fat emulsion-type sausages. The following formulations of the sausages were prepared: pork backfat 20% (PF20); pork backfat 10% + KEG 10% (KEG10); KEG 20% (KEG20); pork backfat 10% + CD-KEG 10% (CD-KEG10); CD-KEG 20% (CD-KEG20); and pork backfat 5% (PF5). The CD-KEG20 formulation exhibited higher viscosity and viscoelasticity than KEG20, which suggested that CD improves the rheological properties and the thermal stability of meat batter. Additionally, CD-KEG20 showed similar emulsion stability, cooking yield and texture parameters compared with PF20. Therefore, 6% CD-added KEG is a suitable fat substitute for preparing low-fat emulsion-type sausages.

## 1. Introduction

The widespread consumption of various types of meat products such as sausages, ham, and bacon can be attributed to their attractive sensory profiles and nutritional value. However, the high levels of fat (approximately 20–30%) and saturated fatty acids in meat products are a major health concern. The consumption of saturated fatty acids is reported to increase the risk of cardiovascular and non-alcoholic fatty liver diseases [1,2]. Thus, recent studies have focused on devising strategies to decrease the content of animal fat in meat products. Several studies have focused on developing fat substitutes in meat products, such as sausages. Among the various types of fat substitutes, emulsion gels and oleogels are representative fat substitutes [3,4]. Emulsion gel, which is a structured emulsion comprising polysaccharides and/or proteins [5], is a potential fat substitute as it can include vegetable oil that contains abundant unsaturated fatty acids [6]. The characteristics of the emulsion gel are determined based on the type or quantity of the gelling agent. Furthermore, the characteristics of emulsion gels directly influence the textural properties and emulsion stability of meat products. Thus, various studies have examined the potential of emulsion gel as a fat substitute in meat products [7,8]. Polysaccharides, such as carrageenan, inulin, and konjac, have advantages as gelling agents due to their high gel-forming ability and gel stability. In recent studies, emulsion gels prepared with carrageenan and zein/carboxymethyl dextrin were employed for fat substitute of sausages [9]. In this study, zein/carboxymethyl dextrin was used to reinforce the crosslinking reaction with carrageenan gel, which has high gelling ability but poor mechanical properties. Other polysaccharides, such as inulin, bamboo fiber, polydextrose, and α-cyclodextrin were also employed to make emulsion gels for a fat substitute in sausages [10]. As shown in these studies, polysaccharides can be appropriate gelling agents and supplementary materials that form bonds with each other in the development of fat replacer emulsion gels.

Konjac glucomannan, a water-soluble, non-ionic polysaccharide, comprises D-glucose and D-mannose at a ratio of 1:1.6 with acetyl groups at the side chain. Alkali treatment promotes the aggregation of konjac molecules through deacetylation and consequently promotes gelation [11]. Konjac-based gel is a potential fat substitute in meat products owing to its physical properties, such as enhanced water-holding capacity and gel strength [12,13]. However, konjac gel cannot easily form stable hydrophobic interactions with oil in emulsion gel, as it mainly comprises hydrogen bonds, which decrease the emulsion stability in meat products. To maintain the stability of emulsion gel, other polysaccharides (such as carrageenan and cornstarch) have been used in konjac-based emulsion gels (KEGs) [14,15].

Cyclodextrin (CD), an enzyme-modified oligosaccharide, can be classified into α-CD, β-CD, and γ-CD based on the number of glucose units (6, 7, and 8 glucose units, respectively) [16]. As CD contains both a hydrophobic cavity and a hydrophilic exterior, it has been used to form complexes in foods to increase their stability, bioavailability, or antioxidant activity [16,17,18]. A recent study demonstrated that CD increased the texture and freeze–thaw stability by forming hydrogen bonds in κ-carrageenan/konjac glucomannan gel [19].

We hypothesized that the supplementation of CD to KEG will promote the formation of hydrophobic bonds with vegetable oil and that of hydrogen bonds with KEG, which will enhance the physicochemical properties of the gel. This study aimed to analyze the physicochemical properties of CD-supplemented KEG (CD-KEG) and examine the potential application of CD-KEG as a fat substitute in low-fat sausages. The gel properties, including bonding variation, microstructure, and textural properties, of CD-KEG were examined. Furthermore, the rheological and textural properties, water holding capacity, and emulsion stability of CD-KEG-supplemented sausages were also analyzed.

## 2. Results and Discussion

### 2.1. Effects of Cyclodextrin on Structural Properties of Emulsion Gels

**Figure 1:**
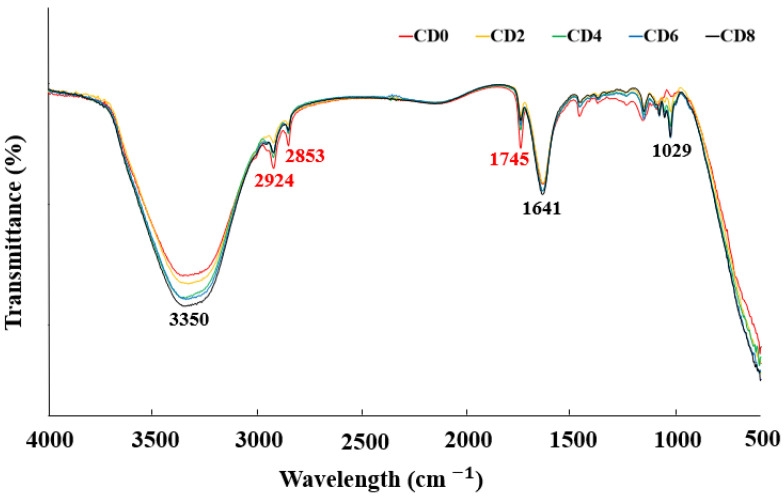
Fourier-transform infrared (FT-IR) spectra of the emulsion gels. The konjac (4%, *w*/*w*) emulsion gels were supplemented with various concentrations of cyclodextrin (CD; 0–8%, *w*/*w*). The peaks detected at wavelengths 3350 and 1641 cm^−1^ indicate O-H stretching, while those detected at 2924 and 2853 cm^−1^ indicate -CH_2_- stretching in long-chain fatty acids. The peaks detected at wavelengths of 1745 and 1029 cm^−1^ indicate C=O stretching in fatty acid esters and C-O stretching vibration in anhydro-glucose units, respectively (*n* = 3). Red, CD0 (0% CD-supplemented konjac-based emulsion gel); yellow, CD2 (2% CD-supplemented konjac-based emulsion gel); green, CD4 (4% CD-supplemented konjac-based emulsion gel); blue, CD6 (6% CD-supplemented konjac-based emulsion gel); black, CD8 (8% CD-supplemented konjac-based emulsion gel).

The effect of CD supplementation on the emulsion gel structure was examined by analyzing the FT-IR spectra. The peaks resulting from chemical interactions between the gelling agents and oil are presented in Figure 1. CD increased the peaks of absorption bands at 3350–3356 and 1641 cm^−1^ in a dose-dependent manner. The increased intensity of these bands, which indicate O-H stretching, suggested an increased number of inter-molecular and intra-molecular hydrogen bonds in the gel [20]. Moreover, CD supplementation shifted the peak from 3356 cm^−1^ (CD0) to 3354 cm^−1^ (CD6) and 3350 cm^−1^ (CD8). A previous study reported that the shifting of the peak to a lower wavelength at 3300–3500 cm^−1^ indicates an increased interaction force resulting from hydrogen bonds [21]. The findings of this study indicate that CD supplementation can reinforce hydrogen bonds in the gel. Hydrogen bonds are essential for the formation of konjac-based gel [22]. Therefore, CD can enhance the sturdiness of the konjac-based gels by reinforcing hydrogen bonds between konjac and CD. Additionally, the intensity of absorption bands at 1029 cm^−1^ in the CD-supplemented group was higher than that in the CD0 group. The bands at 1020–1030 cm^−1^ indicate C-O stretching vibration in anhydro-glucose units [20]. The enhanced numbers of the C-O bond are also related to the amorphous phases of carbohydrates and the alterations of the macromolecular order [23]. Both konjac and CD mostly comprise glucose. Additionally, as CD was deprotonated under alkaline conditions (pH > 12.0), calcium hydroxide solution (pH 12.43) treatment can modify CD [24]. Therefore, the increased intensity of C-O bonds can be attributed to the interaction between konjac and CD. CD supplementation decreased the intensity of absorption bands at 2924 cm^−1^, 2853 cm^−1^, and 1745 cm^−1^, dose-dependently. The bonds at 2924 cm^−1^ and 2853 cm^−1^ result from -CH_2_- stretching, while those at 1745 cm^−1^ result from C=O stretching vibrations [25]. These bonds are related to fat and oil because the -CH_2_- and C=O bonds are included in long-chain fatty acids and fatty acid esters, respectively. The findings of this study indicate that CD supplementation dose-dependently decreased the number of bonds related to oil even though the oil content was identical in all treatment groups. The decreased number of bonds may result from the interaction of oil with CD through the hydrophobic interior of CD. A previous study demonstrated that the C=O bond found in emulsion gel results from the oil residue [26]. Thus, CD supplementation modified the gel structure by increasing the number of hydrogen bonds and hydrophobic interactions with oil, respectively.

### 2.2. Effects of Cyclodextrin on the pH, Color, and TPA of Emulsion Gels

Table 1 shows the pH, color, and texture characteristics of the emulsion gels. The pH values, which were in the range of 7.32–7.36, were not significantly different among the groups (*p* > 0.05). This indicates that the supplementation with CD did not affect the pH of KEGs.

The color attributes of the emulsion gel are important as they are related to emulsion stability and aggregation of oil droplets [27]. Compared with those in the CD-supplemented emulsion gels, the lightness (L*) value and redness (a*) value were significantly lower, and the yellowness (b*) value was significantly higher in the CD0 group (*p* < 0.05). The L* and a* values were not significantly different between the CD-supplemented groups. CD supplementation dose-dependently and significantly decreased the b* values (*p* < 0.05). A high L* value suggested increased light reflection and scattering. The decreased L* value in emulsions was reported to result from the aggregation of oil droplets and large particle size [28,29]. In this study, the increased L* values in CD-supplemented emulsion gel indicated an enhanced emulsion stability. The increased a* value in CD-supplemented groups positively affected the color characteristics of sausages due to consumer preference for a red color in meat products [12]. Meanwhile, CD decreased the b* value dose-dependently, which may be attributed to the color of the CD powder (b* value = −0.46), which was opaque white.

The strength of the gel can directly affect the hardness of low-fat sausages [30]. CD dose-dependently and significantly increased the values of the texture parameters (hardness, springiness, cohesiveness, chewiness, and gumminess) (*p* < 0.05) up to a concentration of 6%. This can be attributed to the effects of hydrogen bonds and C-O bonds, which were observed in the FT-IR spectra. A previous study reported that interactions, including hydrogen bonding, between konjac glucomannan and flaxseed gum enhanced the texture parameter values of a compound gel [31]. Several types of CD were also reported that bind to the surface of the kappa-carrageenan gel through hydrogen bonding, which improves the gel strength [32]. The texture parameter values were not significantly different between the CD6 and CD8 groups (*p* > 0.05). This is consistent with the results of a previous study, which reported that excessive CD content decreases the hardness of the kappa-carrageenan/konjac glucomannan compound gel [19]. These data suggested that high concentrations of sugars in the gel network can inhibit gel formation. Consequently, the CD6 group, which exhibited the highest texture parameter values, was used as a fat substitute to formulate emulsion-type sausages.

### 2.3. Effect of Cyclodextrin on Microstructure of Emulsion Gels

**Figure 2:**
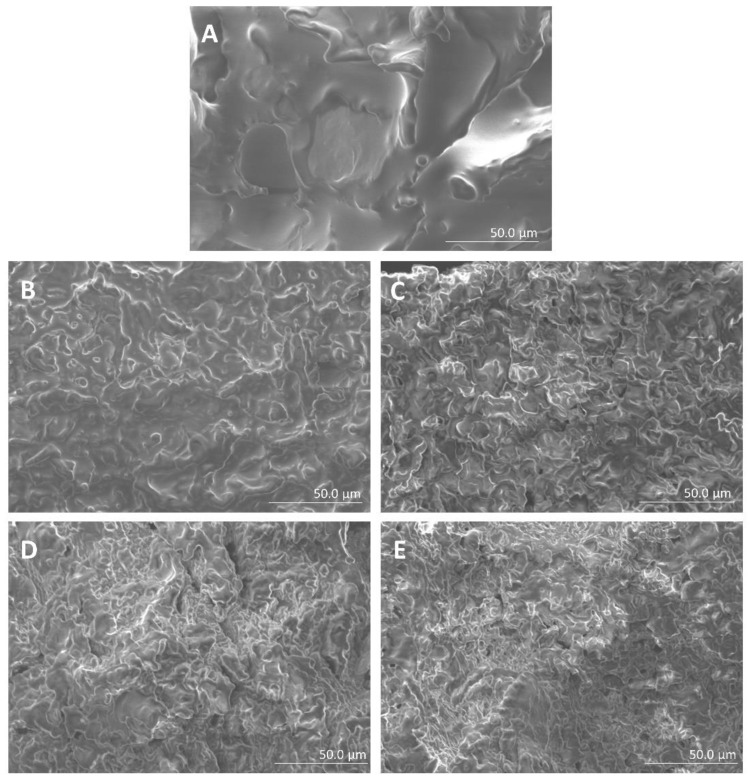
Scanning electron microscopy analysis of the emulsion gels. Scanning electron micrographs of the konjac emulsion gels supplemented with cyclodextrin (CD; 0–8%, *w*/*w*) (magnification = 800×, *n* = 3). (**A**) CD0, 0% CD-supplemented konjac-based emulsion gel; (**B**) CD2, 2% CD-supplemented konjac-based emulsion gel; (**C**) CD4, 4% CD-supplemented konjac-based emulsion gel; (**D**) CD6, 6% CD-supplemented konjac-based emulsion gel; (**E**) CD8, 8% CD-supplemented konjac-based emulsion gel.

The microstructure of the emulsion gels was examined using SEM. Scanning electron micrographs of emulsion gels are shown in Figure 2. The emulsion gel without CD exhibited a smooth and uncomplicated structure; however, supplementation of CD increased the density and compactness of the gel structure. These structural changes can be attributed to the increased number of chemical bonds, and this resulted in the increase in gel strength. A previous study showed that a denser structure of konjac gels with arabic gum was induced by the hydrogen bonds, and this increased the gel strength [33]. Our data showed that the CD6 and CD8 groups exhibited a similarly compact microstructure which was better than that of other groups.

### 2.4. Rheological Properties of Emulsion Gel-Supplemented Meat Batters

**Figure 3:**
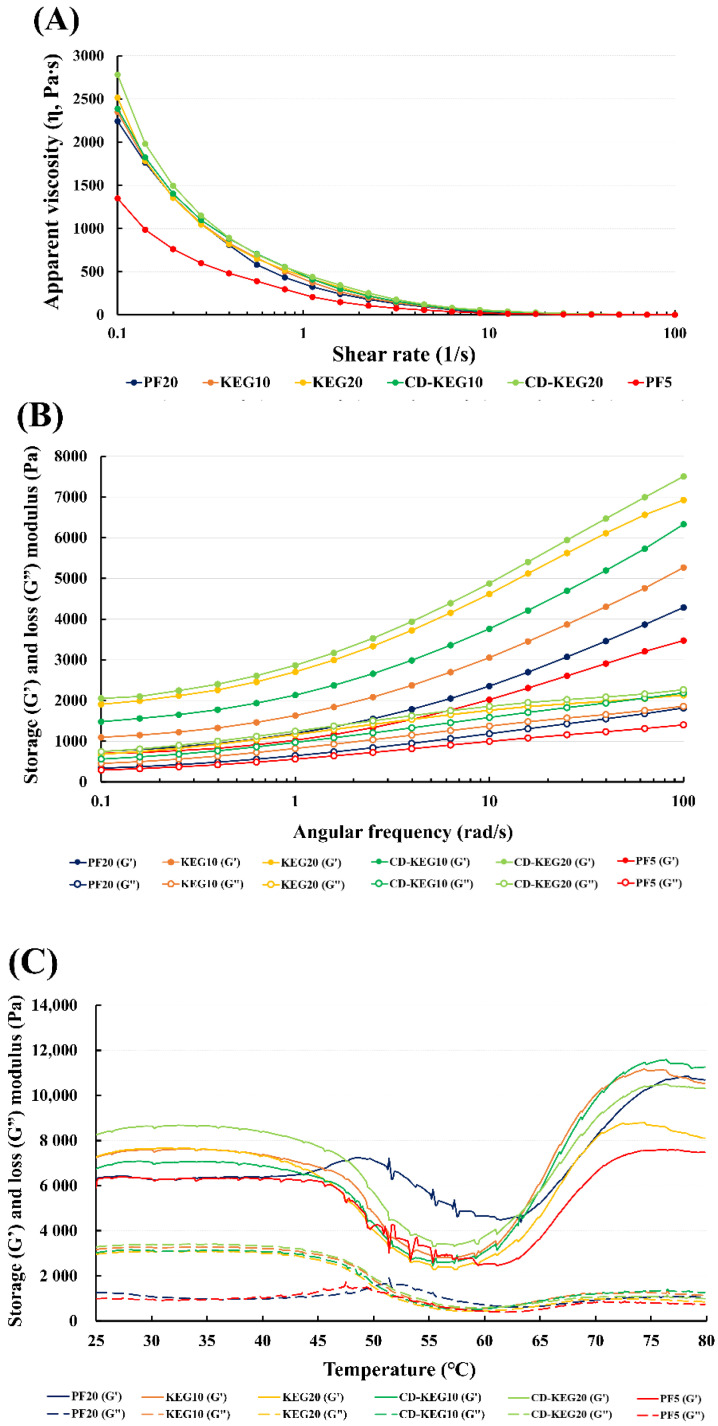
Rheological properties of emulsion gel-supplemented meat batter. (**A**) Apparent viscosity at 10 °C, (**B**) viscoelasticity as a function of frequency in the range of 0.1–100 rad/s at 10 °C, and (**C**) viscoelasticity during temperature sweep at 25–80 °C, 1 Hz, and 0.5% strain. Each colored line represents the rheological properties of the gel-supplemented sausages (*n* = 3). Navy, PF20 (20% pork backfat); orange, KEG10 (10% pork backfat + 10% konjac-based emulsion gel); yellow, KEG20 (20% konjac-based emulsion gel), green, CD-KEG10 (10% pork backfat + 10% cyclodextrin (CD)-supplemented konjac-based emulsion gel); light green, CD-KEG20 (20% CD-supplemented konjac-based emulsion gel); red, PF5 (5% pork backfat).

The apparent viscosity of the meat batters is presented in Figure 3A. Meat batters of CD-KEG10 and CD-KEG20 were formulated with 6% CD-supplemented KEGs. Additionally, formulations containing only KEGs or pork backfat were prepared. PF5 group was prepared as a low-fat sausage control. The apparent viscosity was measured at 10 °C based on the batter processing conditions. The CD-KEG20 group exhibited the highest viscosity at all shear rates (Figure 3A). The viscosity values of CD-KEG10 and CD-KEG20 meat batters were higher than those of KEG10 and KEG20 meat batters. This was affected by the high texture profiles of CD-KEG (Table 1). The PF20 group exhibited lower viscosity values than the CD-KEG and KEG groups. The PF5 group exhibited the lowest viscosity. Fat content affects the viscosity and texture of meat products, and high viscosity was related to the increment of emulsion stability [34]. Hence, pork backfat can be substituted with KEGs, especially CD-KEG20, in meat products.

The viscoelasticity data of the meat batters obtained at 10 °C are shown in Figure 3B. This temperature during the frequency sweep was also selected based on the batter processing conditions. All the samples exhibited higher G′ values than G″ values, which indicated that meat batters exhibited gel-like properties. The viscoelasticity data during the frequency sweep showed a similar tendency to the apparent viscosity data. In a previous study, reduced-fat sausages had the lowest viscoelasticity [6]. However, the supplementation of meat batters with emulsion gels (KEG and CD-KEG) increased the viscoelasticity during the frequency sweep.

The G′ and G″ values of the meat batter at 25–80 °C are presented in Figure 3C. The heating process resulted in protein denaturation and gelation in meat products. Hence, the viscoelasticity data during temperature sweep were useful for evaluating heat-induced changes. The G′ values of all samples changed during heating. The decreased G’ value at temperatures up to 55–60 °C can be attributed to the denaturation and unfolding of myosin. At temperatures higher than 60 °C, denatured and unfolded protein residues interact with adjacent proteins, which leads to aggregation and gel network formation [35]. In this study, the viscoelasticity of the CD-KEG20 group was higher than that of the KEG20 group at temperatures higher than 60 °C. Additionally, the viscoelasticity of the CD-KEG20 group was similar to that of the PF20 group. These data indicate that CD promotes the interaction between denatured proteins and emulsion gels, which results in the formation of a reinforced gel network with increased thermal stability.

### 2.5. Emulsion Stability and Water Holding Capacity of Emulsion Gel-Supplemented Meat Batter and Cooking Yield of Emulsion Gel-Supplemented Sausages

**Figure 4:**
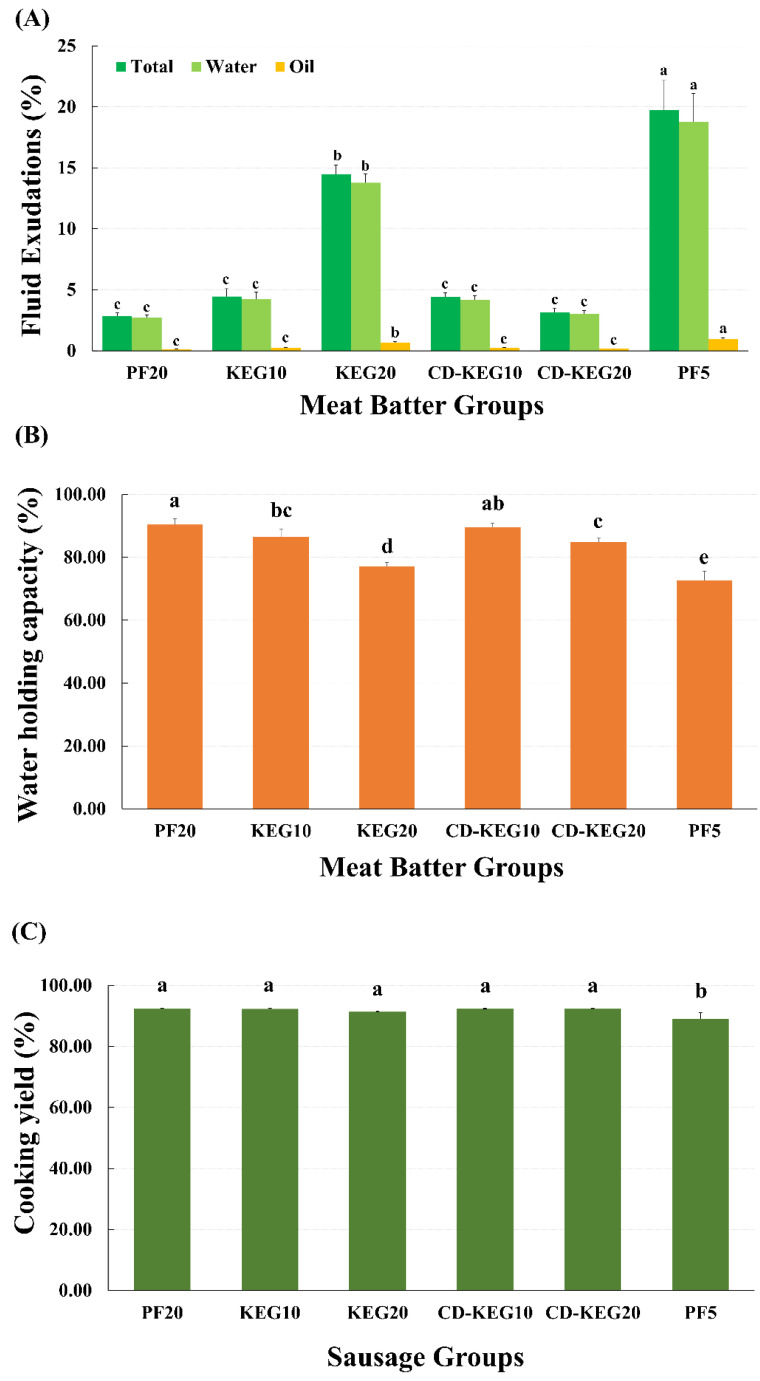
Emulsion stability and water holding capacity of emulsion gel-supplemented meat batter and cooking yield of emulsion gel-supplemented sausages. (**A**) Emulsion stability of meat batter, (**B**) water holding capacity of meat batter, and (**C**) cooking yield of sausages were evaluated. Emulsion stability is presented by the percentage of fluid exudation during heating. (**A**) Green, total fluid exudation (%); light green, water fluid exudation (%); orange, oil fluid exudation (%). PF20, 20% pork fat; KEG10, 10% pork fat + 10% konjac-based emulsion gel; KEG20, 20% konjac-based emulsion gel; CD-KEG10, 10% pork fat + 10% cyclodextrin (CD)-supplemented konjac-based emulsion gel; CD-KEG20, 20% CD-supplemented konjac-based emulsion gel; PF5, 5% pork fat. ^a–e^ Different letters represent a significant difference in each fluid exudation and cooking yield (*p* < 0.05).

The emulsion stability of emulsion gel-supplemented meat batter is shown in Figure 4A. The emulsion stability of sausages was measured based on the release of total fluids, water, and oil after the application of centrifugation and heat treatment. The emulsion stability parameters were not significantly different between the CD-KEG20 and PF20 groups (*p* > 0.05). These data suggest that CD-KEG can improve emulsion stability in low-fat emulsion-type sausages. The emulsion stability of the KEG20 group was significantly lower than that of the CD-KEG20 group (*p* < 0.05). This suggested that CD did not only bond with water and oil in the KEG, also interacted with water and oil in the meat batter. Thus, the supplementation of KEG with CD improves emulsion stability of the meat batter.

The water holding capacity (WHC) of the meat batters is presented in Figure 4B. The WHC of the meat batters was improved by the use of CD-KEG as a pork backfat substitute, compared with KEG without CD. Addition of CD to KEG reinforced hydrogen bonds in the gels and improved rheological properties of meat batters, as shown in Figure 1 and Figure 3. It seems that CD can form hydrogen bonds with surrounding water molecules in meat batter. Furthermore, the increased viscosity and viscoelasticity of the CD-KEG group might have decreased the movement of water and fat droplets. In a previous study, the addition of the β-CD/ovalbumin mixture in myofibrillar protein gel increased the water holding capacity of the gel during the storage time [36]. Collectively, the addition of CD to KEG can increase the WHC of the meat batter.

The cooking yield was not significantly different between the groups, except for the PF5 group (Figure 4C). The PF5 group exhibited decreased emulsion stability (increased release of total fluids, water, and oil), which was associated with decreased cooking yield. Therefore, our data suggest that the addition of emulsion gel to low-fat sausages enhanced the cooking yield, and CD also can improve emulsion stability.

### 2.6. pH, Color, and TPA of Emulsion-Type Sausages

The pH, color and TPA of emulsion-type sausages are shown in Table 2. The pH values of sausages were in the range of 6.08–6.18. Mostly, the pH values were not significantly different among different groups. However, the pH value of the CD-KEG20 group was higher than that of the PF20 group (*p* < 0.05). The pH of the emulsion-type sausages may be affected by the pH of CD-KEG (pH 7.36). This explains the high pH of the CD-KEG20 group, which contains the highest amount of CD-KEG (20%). Like our data, it was reported that the pH of fat substitute could affect the pH of sausages, where low pH of konjac gel with cactus pear powder decreased the pH of frankfurter-type sausage [12].

Among the color parameters, the L* values of the KEG-supplemented and CD-KEG-supplemented groups were lower than those of the PF20 group. This suggests that the supplementation of sausage with a konjac-based gel decreased the L* value. A previous study reported that the supplementation of konjac gel for merguez sausage decreased the L* value [37]. The a* and b* values of the KEG10, KEG20, CD-KEG10, and CD-KEG20 groups were generally higher than those of the PF20 group. Substitution of pork backfat as oil increased the b* value in a previous study [38]. These findings indicate that supplementation with KEG or CD-KEG affected the color parameters of sausages.

TPA revealed that the texture characteristics of the PF20, KEG10, KEG20, CD-KEG10, and CD-KEG20 groups were better than those of the PF5 group (*p* < 0.05). Low fat contents in meat products can decrease the hardness value [8,30,39]. The texture values of CD-KEG10 were highest, and were similar between the PF20 and CD-KEG20 groups. Additionally, the CD-KEG10 and CD-KEG20 groups exhibited better texture characteristics than the KEG10 and KEG20 groups. These findings indicate that CD-KEG can be used as a pork backfat substitute in emulsion-type sausages without adversely affecting the texture quality.

## 3. Conclusions

This study investigated the physicochemical properties of CD-KEGs and evaluated the effect of CD-KEG supplementation as a fat substitute on the quality characteristics of sausages. The supplementation of KEG with CD improved the textural properties and structural density of the gel by increasing the number of hydrogen bonds and hydrophobic bonds. In particular, 6% CD supplementation was optimal for preparing CD-KEG. Among the different sausage groups, the CD-KEG20 (supplemented with 0% pork backfat and 20% CD-KEG) exhibited comparable rheological properties, emulsion stability, cooking yield, and texture characteristics compared with the PF20 (supplemented with 20% pork backfat). The KEG20 (supplemented with 0% pork backfat and 20% KEG without CD) exhibited increased thermal stability, emulsion stability, water holding capacity, and texture compared with PF5 (supplemented with 5% pork backfat); however, it could not reach the level of PF20. This suggested that CD plays an important role in improving the stability and texture of KEGs. Thus, this study demonstrated that CD enhanced the properties of konjac-based gels, and that CD-KEG can partially or completely replace pork backfat in emulsion-type sausages.

## 4. Materials and Methods

### 4.1. Materials

Konjac glucomannan and β-CD were purchased from Yujin Co. (Gyeonggi, Korea) and Serimfood (Bucheon, Korea), respectively. Canola oil was purchased from CJ Cheiljedang (Seoul, Korea). Pork lean meat from semitendinosus, semimembranosus, and biceps femoris muscles and backfat were purchased from the Eumseong Nonghyup (Eumseong, Korea). Calcium hydroxide and other analytical materials were purchased from Sigma-Aldrich (St. Louis, MO, USA).

### 4.2. Preparation of Emulsion Gels

Gels were prepared following the protocols of a previous study with minor modifications (Table 3) [40]. Briefly, konjac powder (4%, *w*/*w*) was homogenized with distilled water at 3000 rpm for 5 min using a bowl cutter (C4 VV, Sirman, Venezia, Italy). Next, the homogenate was supplemented with various concentrations (0%, 2%, 4%, 6%, and 8% (*w*/*w*)) of CD. The mixture was homogenized at 3000 rpm for 5 min. Further, canola oil (20%; *w*/*w*) was added to the konjac-CD solution and emulsified at 3000 rpm for 5 min. The emulsified mixture was cooled to 10 °C because the increased temperature of gels caused by the emulsification process can lead to excessively rapid gelation during alkali treatment. Then, the mixture was gently mixed with 1% calcium hydroxide solution (10%; *w*/*w*) at 1500 rpm for 3 min. CD-supplemented konjac-based emulsion gels were compressed vertically using a burger press for 10 s to remove bubbles in the gels and obtain uniform-sized gels for texture analysis. The gels were stored at 4 °C for 24 h before analysis.

### 4.3. Fourier-Transform Infrared (FT-IR) Spectroscopy of Emulsion Gels

The bonding transition of the emulsion gels was measured by means of an FT-IR spectrophotometer (FT/IR-4700, JASCO, Tokyo, Japan) using the method of [41]. The spectral data were recorded under the following conditions: wavelength, 500–4000 cm^−1^; resolution, 1 cm^−1^. The gels used for FT-IR were cut into cubes and analyzed in triplicate.

### 4.4. pH and Color of Emulsion Gels

The pH values of the samples were measured in triplicate using a LAQUA pH meter (Horiba, Kyoto, Japan). To measure the pH, 5 g of the sample was homogenized in 20 mL of distilled water. The color of the emulsion gel surface was measured in seven replicates using a CR-210 colorimeter (Konica Minolta, Ltd., Osaka, Japan). The data are presented as L* (lightness), a* (redness), and b* (yellowness) values according to the International Commission on Illumination. Before measurement, calibration of colorimeter was conducted with a white plate (L* = +97.27, a* = +5.21, b* = −3.40).

### 4.5. Texture Profile Analysis (TPA) of Emulsion Gels

TPA of the gel was performed in five replicates using a texture analyzer (TA-XT2i, Stable Micro Systems Ltd., England) equipped with a spherical probe (5 mm of diameter). The gels were cut into squares (with length 2.5 cm) and the texture of gels was measured under the following conditions: distance, 20.0 mm; pre-test speed, 2.0 mm/s; test speed, 1.0 mm/s; post-test speed, 3.0 mm/s; force, 5 g.

### 4.6. Microstructure of Emulsion Gels

The microstructure of the emulsion gels was examined using a scanning electron microscope (SEM, su8010, Hitachi, Tokyo, Japan). The emulsion gels were lyophilized using a vacuum freeze dryer at −80 °C for 48 h (MCFD 8508, Il Shin Bio, Yangju, Korea). The lyophilized samples were coated with platinum using an ion sputter (MC1000, Hitachi, Tokyo, Japan). The conditions for SEM were as follows: acceleration voltage, 5 kV; magnification, 800×.

### 4.7. Preparation of Emulsion Gel-Supplemented Meat Batter and Sausages

To verify the replaceability of pork backfat with KEG and CD-KEG, the following six formulations of meat batter and sausages were prepared (Table 4): pork fat 20% (PF20); pork fat 10% + KEG 10% (KEG10); KEG 20% (KEG20); pork fat 10% + CD-KEG 10% (CD-KEG10); CD-KEG 20% (CD-KEG20); pork fat 5% (PF5). All other ingredients were identically added to meat batter and the sausages. To process the sausages, trimmed lean meat and pork backfat were minced through a 3 mm plate using a mincer (PM-70, Mainca, Barcelona, Spain). All of the ingredients, including minced meat, backfat and emulsion gels, were put into the bowl cutter (C4 VV, Sirman, Venezia, Italy) and emulsified for 4 min. The emulsified batter was stuffed into a collagen casing (240 mm, NIPPI Inc., Tokyo, Japan) using a stuffer (IS-8, Sirman, Marsango, Italy). The sausages were cooked in a smokehouse at 80 °C for 30 min to allow the core temperature to reach 72 °C. All sausages were cooled to room temperature (25 ± 1 °C) and stored at 4 °C.

### 4.8. Rheological Properties of Emulsion Gel-Supplemented Meat Batter

Rheological properties (apparent viscosity and viscoelasticity) were measured using a rheometer (MCR 92, Anton Paar, Graz, Austria) equipped with a 25 mm-diameter parallel plate. Meat batters were loaded on a static plate, and a 1 mm gap was maintained between the plates. The excess sample was trimmed, and the periphery of the plate was covered with oil to prevent water evaporation from the sample during the test. The apparent viscosity was measured at 10 °C with shear rates of 0.1–100 1/s in triplicate. The linear viscoelasticity region (LVR) was confirmed by performing strain sweep tests under the following conditions: strain, 0.01%–10%; frequency, 1 Hz. The frequency sweep test was performed under the following conditions: angular frequency, 0.1–100 rad/s; temperature, 10 °C; strain, 0.5% (within LVR). The temperature sweep test was performed under the following conditions to examine the effect of heating on the batter: heating rate, 2 °C/min; temperature, 25–80 °C; frequency, 1 Hz; strain, 0.5%. At each stage, the storage modulus (G′) and loss modulus (G″) were measured, and measurement was performed in triplicate.

### 4.9. Emulsion Stability of Emulsion Gel-Supplemented Meat Batter

The emulsion stability of meat batters was measured as previously reported by [42], with minor modifications. The meat batter (25 g) was filled in a conical tube and centrifuged at 5000× *g* for 5 min at 10 °C. Then, the samples were heated in a water bath at 80 °C for 30 min and cooled to room temperature (25 ± 1 °C). The liquid released from the meat batter was transferred to a pre-weighed aluminum weighing dish. The dishes containing the liquid were weighed and placed in a dry oven at 105 °C for 24 h. Next, the dishes were cooled to room temperature (25 ± 1 °C) and weighed again. Total, water, and fat fluid exudations were calculated using the following equations:

Total fluid exudation (%) = (weight of initial released liquid (g)/weight of meat batter (g)) × 100(1)

Water exudation (%) = [(weight of initial released liquid + weight of dish (g) − weight of the dish after drying (g))/weight of meat batter (g)] × 100(2)

Fat exudation (%) = [(weight of the dish after drying (g) − weight of the empty dish (g))/weight of meat batter (g)] × 100(3)

### 4.10. Water Holding Capacity (WHC) of Emulsion Gel-Supplemented Meat Batter

The WHC of meat batters was determined as described previously [43]. The meat batter (10 g) was filled in a tube and centrifuged at 6000× *g* for 15 min at 10 °C. After the centrifugation, the samples were heated in a water bath for 15 min at 85 °C and cooled to room temperature (25 ± 1 °C). The cooled samples were centrifuged again at 6000× *g* for 15 min at 10 °C. WHC was obtained using the following equation:

WHC (%) = [(weight of the meat batter after heating and centrifugation (g)/weight of the meat batter (g)] × 100(4)

### 4.11. Cooking Yield of Emulsion Gel-Supplemented Sausages

The meat emulsion stuffed in the casing was weighed, and the samples were heated at 80 °C for 30 min. The cooked samples were cooled to room temperature and weighed. The cooking yield was calculated using the following equation:

Cooking yield (%) = (weight of a cooked meat emulsion (g)/weight of a stuffed raw meat emulsion (g)) × 100(5)

### 4.12. pH, Color and TPA of Emulsion Gel-Supplemented Sausages

The pH, color, and TPA were measured as described in Section 4.4 and Section 4.5. The color of the sausage was measured on sliced surface. The sausage for TPA was prepared to a size of 2.5 cm (length) × 2.5 cm (diameter).

### 4.13. Statistical Analysis

Data are presented as mean ± standard deviation using at least triplicate per group. All statistical analyses were performed using SPSS-PASW Ver. 18.0 (SPSS Inc., Chicago, IL, USA). The means were analyzed using one-way analysis of variance (ANOVA), followed by Duncan’s multiple range post hoc test. Differences were considered significant at *p* < 0.05.

## Figures and Tables

**Table 1:** pH, color, and texture parameters of emulsion gels.

Parameters		CD-Supplemented Konjac-Based Emulsion Gels ^(1)^				
		CD0	CD2	CD4	CD6	CD8
pH		7.32 ± 0.02	7.36 ± 0.03	7.36 ± 0.04	7.36 ± 0.02	7.34 ± 0.04
CIE	L*	72.96 ± 1.41 ^b^	92.18 ± 0.40 ^a^	92.35 ± 0.43 ^a^	92.32 ± 0.34 ^a^	92.33 ± 0.45 ^a^
	a*	0.74 ± 0.18 ^b^	1.19 ± 0.05 ^a^	1.20 ± 0.05 ^a^	1.20 ± 0.07 ^a^	1.26 ± 0.06 ^a^
	b*	8.61 ± 0.23 ^a^	5.68 ± 0.15 ^b^	5.44 ± 0.22 ^c^	5.19 ± 0.20 ^d^	4.79 ± 0.19 ^e^
Hardness (g)		95.46 ± 12.08 ^c^	220.56 ± 4.27 ^b^	239.00 ± 21.48 ^b^	267.98 ± 13.76 ^a^	268.42 ± 21.07 ^a^
Springiness		0.92 ± 0.01 ^b^	0.93 ± 0.01 ^ab^	0.94 ± 0.01 ^ab^	0.94 ± 0.02 ^a^	0.95 ± 0.02 ^a^
Cohesiveness		0.29 ± 0.03 ^d^	0.37 ± 0.02 ^c^	0.38 ± 0.02 ^bc^	0.39 ± 0.01 ^ab^	0.41 ± 0.00 ^a^
Chewiness (g)		23.03 ± 4.87 ^d^	69.27 ± 6.37 ^c^	81.52 ± 8.05 ^b^	94.24 ± 5.11 ^a^	98.07 ± 2.99 ^a^
Gumminess (g)		28.66 ± 4.86 ^d^	74.86 ± 7.51 ^c^	91.16 ± 10.44 ^b^	100.25 ± 4.17 ^a^	105.77 ± 4.92 ^a^

CIE, Commission Internationale de l’Eclairage. ^(1)^ CD0, 0% cyclodextrin-supplemented konjac-based emulsion gel; CD2, 2% cyclodextrin-supplemented konjac-based emulsion gel; CD4, 4% cyclodextrin-supplemented konjac-based emulsion gel; CD6, 6% cyclodextrin-supplemented konjac-based emulsion gel; CD8, 8% cyclodextrin-supplemented konjac-based emulsion gel. Data are presented as mean ± standard deviation (*n* ≥ 3). For ^a–e^ in each row, different letters represent a significant difference (*p* < 0.05).

**Table 2:** pH, color, and texture parameters of emulsion gel-supplemented sausages.

Parameters		Sausage Groups ^(1)^					
		PF20	KEG10	KEG20	CD-KEG10	CD-KEG20	PF5
pH		6.08 ± 0.03 ^b^	6.09 ± 0.02 ^b^	6.14 ± 0.06 ^ab^	6.12 ± 0.08 ^ab^	6.18 ± 0.01 ^a^	6.14 ± 0.02 ^ab^
CIE	L*	82.05 ± 0.52 ^a^	80.84 ± 0.11 ^b^	79.56 ± 0.46 ^c^	81.18 ± 0.36 ^b^	79.07 ± 0.12 ^d^	81.69 ± 0.32 ^a^
	a*	2.29 ± 0.03 ^c^	2.86 ± 0.02 ^ab^	2.90 ± 0.05 ^a^	2.76 ± 0.03 ^b^	2.93 ± 0.19 ^a^	2.21 ± 0.03 ^c^
	b*	10.68 ± 0.09 ^b^	11.13 ± 0.12 ^a^	11.14 ± 0.16 ^a^	10.74 ± 0.12 ^b^	11.02 ± 0.09 ^a^	11.21 ± 0.20 ^a^
Hardness (g)		364.45 ± 4.68 ^bc^	354.17 ± 15.31 ^c^	334.50 ± 6.22 ^d^	387.73 ± 8.55 ^a^	369.05 ± 17.56 ^b^	185.63 ± 6.65 ^e^
Springiness		0.98 ± 0.00 ^a^	0.97 ± 0.02 ^a^	0.95 ± 0.01 ^b^	0.97 ± 0.01 ^a^	0.96 ± 0.01 ^ab^	0.90 ± 0.02 ^c^
Cohesiveness		0.43 ± 0.01 ^ab^	0.44 ± 0.02 ^a^	0.41 ± 0.01 ^b^	0.43 ± 0.01 ^ab^	0.43 ± 0.01 ^a^	0.37 ± 0.02 ^c^
Chewiness (g)		151.40 ± 2.02 ^b^	148.89 ± 9.72 ^b^	131.62 ± 4.71 ^c^	161.72 ± 7.10 ^a^	155.03 ± 8.18 ^ab^	62.00 ± 4.59 ^d^
Gumminess (g)		153.64 ± 1.15 ^a^	154.46 ± 7.33 ^a^	140.86 ± 4.04 ^b^	159.77 ± 9.99 ^a^	155.03 ± 6.90 ^a^	75.28 ± 8.51 ^c^

CIE, Commission Internationale de l’Eclairage. ^(1)^ PF20, 20% pork fat; KEG10, 10% pork fat + 10% konjac-based emulsion gel; KEG20, 20% konjac-based emulsion gel; CD-KEG10, 10% pork fat + 10% cyclodextrin (CD)-supplemented konjac-based emulsion gel; CD-KEG20, 20% CD-supplemented konjac-based emulsion gel; PF5, 5% pork fat. Data are presented as mean ± standard deviation (*n* ≥ 3). For ^a–e^ in each row, different letters representing a significant difference (*p* < 0.05).

**Table 3:** The formulation of emulsion gels.

Ingredients (%)	CD-Containing Konjac-Based Emulsion Gels ^(1)^				
	CD0	CD2	CD4	CD6	CD8
Konjac	4	4	4	4	4
β-cyclodextrin	0	2	4	6	8
Distilled water	66	64	62	60	58
Calcium hydroxide (1%)	10	10	10	10	10
Canola oil	20	20	20	20	20
Total	100	100	100	100	100

^(1)^ CD0 (0% cyclodextrin-containing konjac-based emulsion gel), CD2 (2% cyclodextrin-containing konjac-based emulsion gel), CD4 (4% cyclodextrin-containing konjac-based emulsion gel), CD6 (6% cyclodextrin-containing konjac-based emulsion gel), CD8 (8% cyclodextrin-containing konjac-based emulsion gel).

**Table 4:** The formulation of emulsion gel-supplemented sausages.

Ingredients (%)	Sausage Groups ^(1)^					
	PF20	KEG10	KEG20	CD-KEG10	CD-KEG20	PF5
Lean meat	60	60	60	60	60	60
Pork backfat	20	10	0	10	0	5
KEG	0	10	20	0	0	0
CD-KEG	0	0	0	10	20	0
Ice	20	20	20	20	20	35
Total	100	100	100	100	100	100
Salt	1.5	1.5	1.5	1.5	1.5	1.5
Sodium tripolyphosphate	0.3	0.3	0.3	0.3	0.3	0.3

KEG, konjac-based emulsion gel without CD; CD-KEG, CD-supplemented konjac-based emulsion gels. ^(1)^ PF20, 20% pork fat; KEG10, 10% pork fat + 10% konjac-based emulsion gel; KEG20, 20% konjac-based emulsion gel; CD-KEG10, 10% pork fat + 10% CD-containing konjac-based emulsion gel; CD-KEG20, 20% CD-containing konjac-based emulsion gel; PF5, 5% pork fat.

## Data Availability

Data are contained within the article.
